# Job strain in relation to body mass index: pooled analysis of 160 000 adults from 13 cohort studies

**DOI:** 10.1111/j.1365-2796.2011.02482.x

**Published:** 2011-12-05

**Authors:** S T Nyberg, K Heikkilä, E I Fransson, L Alfredsson, D De Bacquer, J B Bjorner, S Bonenfant, M Borritz, H Burr, A Casini, E Clays, N Dragano, R Erbel, G A Geuskens, M Goldberg, W E Hooftman, I L Houtman, K-H Jöckel, F Kittel, A Knutsson, M Koskenvuo, C Leineweber, T Lunau, I E H Madsen, L L Magnusson Hanson, M G Marmot, M L Nielsen, M Nordin, T Oksanen, J Pentti, R Rugulies, J Siegrist, S Suominen, J Vahtera, M Virtanen, P Westerholm, H Westerlund, M Zins, J E Ferrie, T Theorell, A Steptoe, M Hamer, A Singh-Manoux, G D Batty, M Kivimäki, for the IPD-Work Consortium

**Affiliations:** 1 From the Finnish Institute of Occupational HealthHelsinki, Finland; 2 Institute of Environmental Medicine, Karolinska InstitutetStockholm; 3 School of Health Sciences, Jönköping UniversityJönköping, Sweden; 4 Department of Public Health, Ghent UniversityGhent, Belgium; 5 National Research Centre for the Working EnvironmentCopenhagen, Denmark; 6 Versailles-Saint Quentin UniversityVersailles; 7 Inserm U1018, Centre for Research in Epidemiology and Population HealthVillejuif, France; 8 Department of Occupational Medicine, Bispebjerg University HospitalCopenhagen, Denmark; 9 Centre for Maritime Health and SafetyEsbjerg, Denmark; 10 School of Public Health, Université Libre de BruxellesBrussels, Belgium; 11 Institute for Medical Informatics, Biometry, and Epidemiology, University Duisburg-EssenEssen, Germany; 12 Department of Cardiology, West-German Heart Center Essen, University Duisburg-EssenEssen, Germany; 13 TNOHoofddorp, the Netherlands; 14 Department of Health Sciences, Mid Sweden UniversitySundsvall, Sweden; 15 Department of Public Health, University of HelsinkiHelsinki, Finland; 16 Stress Research Institute, Stockholm UniversityStockholm, Sweden; 17 Epidemiology and Public Health, University College LondonLondon, UK; 18 Department of Occupational and Environmental Medicine, Bispebjerg University HospitalCopenhagen, Denmark; 19 Department of Public Health and Clinical Medicine, Occupational and Environmental Medicine, Umeå UniversityUmeå, Sweden; 20 Finnish Institute of Occupational HealthTurku, Finland; 21 Department of Society, Human Development and Health, Harvard School of Public HealthBoston, MA, USA; 22 Department of Public Health and Department of Psychology, University of CopenhagenCopenhagen, Denmark; 23 Department of Medical Sociology, University of DüsseldorfDüsseldorf, Germany; 24 Department of Public Health, University of TurkuTurku; 25 Folkhälsan Research CenterHelsinki; 26 Turku University HospitalTurku, Finland; 27 Occupational and Environmental Medicine, Uppsala UniversityUppsala; 28 Department of Clinical Neuroscience, Karolinska InstitutetStockholm, Sweden; 29 School of Community and Social Medicine, University of BristolBristol; 30 Centre for Cognitive Ageing and Cognitive Epidemiology, University of EdinburghEdinburgh, UK; 31 Department of Behavioral Sciences, University of HelsinkiHelsinki, Finland

**Keywords:** body mass index, cohort studies, job strain, obesity, thinness, work stress

## Abstract

Nyberg ST, Heikkilä K, Fransson EI, Alfredsson L, De Bacquer D, Bjorner JB, Bonenfant S, Borritz M, Burr H, Casini A, Clays E, Dragano N, Erbel R, Geuskens GA, Goldberg M, Hooftman WE, Houtman IL, Jöckel K-H, Kittel F, Knutsson A, Koskenvuo M, Leineweber C, Lunau T, Madsen IEH, Magnusson Hanson LL, Marmot MG, Nielsen ML, Nordin M, Oksanen T, Pentti J, Rugulies R, Siegrist J, Suominen S, Vahtera J, Virtanen M, Westerholm P, Westerlund H, Zins M, Ferrie JE, Theorell T, Steptoe A, Hamer M, Singh-Manoux A, Batty GD, Kivimäki M, for the IPD-Work Consortium (Finnish Institute of Occupational Health, Helsinki, Finland; Institute of Environmental Medicine, Karolinska Institutet, Stockholm, Sweden; Jönköping University, Jönköping, Sweden; Ghent University, Ghent, Belgium; National Research Centre for the Working Environment, Copenhagen, Denmark; Versailles-Saint Quentin University, Versailles, France; Centre for Research in Epidemiology and Population Health, Villejuif, France; Bispebjerg University Hospital, Copenhagen, Denmark; Centre for Maritime Health and Safety, Esbjerg, Denmark; Université Libre de Bruxelles, Brussels, Belgium; University Duisburg-Essen, Essen, Germany; West-German Heart Center Essen, University Duisburg-Essen, Essen, Germany; TNO, Hoofddorp, the Netherlands; Mid Sweden University, Sundsvall, Sweden; University of Helsinki, Helsinki, Finland; Stockholm University, Stockholm, Sweden; University College London, London, UK; Bispebjerg University Hospital, Copenhagen, Denmark; Umeå University, Umeå, Sweden; Finnish Institute of Occupational Health, Turku, Finland; Harvard School of Public Health, Boston, MA, USA; University of Copenhagen, Copenhagen, Denmark; University of Düsseldorf, Düsseldorf, Germany; University of Turku, Turku; Folkhälsan Research Center, Helsinki; Turku University Hospital, Turku, Finland; Uppsala University, Uppsala; Karolinska Institutet, Stockholm, Sweden; University of Bristol, Bristol; University of Edinburgh, Edinburgh, UK; and University of Helsinki, Helsinki, Finland). Job strain in relation to body mass index: pooled analysis of 160 000 adults from 13 cohort studies. *J Intern Med* 2012; **272**: 65–73.

**Background:**

Evidence of an association between job strain and obesity is inconsistent, mostly limited to small-scale studies, and does not distinguish between categories of underweight or obesity subclasses.

**Objectives:**

To examine the association between job strain and body mass index (BMI) in a large adult population.

**Methods:**

We performed a pooled cross-sectional analysis based on individual-level data from 13 European studies resulting in a total of 161 746 participants (49% men, mean age, 43.7 years). Longitudinal analysis with a median follow-up of 4 years was possible for four cohort studies (*n* = 42 222).

**Results:**

A total of 86 429 participants were of normal weight (BMI 18.5–24.9 kg m^−2^), 2149 were underweight (BMI < 18.5 kg m^−2^), 56 572 overweight (BMI 25.0–29.9 kg m^−2^) and 13 523 class I (BMI 30–34.9 kg m^−2^) and 3073 classes II/III (BMI ≥ 35 kg m^−2^) obese. In addition, 27 010 (17%) participants reported job strain. In cross-sectional analyses, we found increased odds of job strain amongst underweight [odds ratio 1.12, 95% confidence interval (CI) 1.00–1.25], obese class I (odds ratio 1.07, 95% CI 1.02–1.12) and obese classes II/III participants (odds ratio 1.14, 95% CI 1.01–1.28) as compared with participants of normal weight. In longitudinal analysis, both weight gain and weight loss were related to the onset of job strain during follow-up.

**Conclusions:**

In an analysis of European data, we found both weight gain and weight loss to be associated with the onset of job strain, consistent with a ‘U’-shaped cross-sectional association between job strain and BMI. These associations were relatively modest; therefore, it is unlikely that intervention to reduce job strain would be effective in combating obesity at a population level.

## Introduction

Obesity and job strain (i.e. stress at work) are major public health issues in modern societies, potentially contributing to a range of health-related outcomes, such as reduced quality of life, disability, cardiovascular and cerebrovascular diseases and depression [1–3]. According to recent European Union estimates, stress is cited as a factor in half of all lost working days and thus represents a substantial cost in terms of human distress and impaired economic performance [4]. There may be a link between job strain and body mass index (BMI) [5–12] – the most commonly utilized measure of adiposity – as stress might contribute to an unhealthy lifestyle [5], such as physical inactivity [6] and a poor diet [7], which in turn could induce weight gain. Other mechanisms are also plausible. Conversely, psychosocial stress may reduce appetite leading to weight loss [8–10]. In addition to stress being a risk factor for weight change, there is a suggestion that this relationship might be bi-directional. Obesity, for instance, may reduce work capacity [11], increasing the risk of feelings of stress (the reverse causation hypothesis). Finally, given its association with both increased weight and exposure to stressful work conditions [12], it is also likely that socio-economic disadvantage may have an important role in these relationships (the common cause hypothesis).

To date, empirical evidence for an association between job strain (or other forms of work stress) and BMI has been inconsistent, with findings of a positive (more stress, higher BMI) [8, 13–17], an inverse (more stress, lower BMI) [18, 19] or no relation [20, 21]. Small sample sizes in most of these studies may have contributed to the mixed results. This low study power has also led to an inability to distinguish between categories of underweight or different classes of obesity. To enable more precise characterization of the association between job strain and BMI than in previous studies, we pooled data from 13 independent cohort studies, resulting in an individual-level meta-analysis of 161 746 men and women.

## Subjects and methods

### Study population and design

This study is part of the individual-participant-data meta-analysis in working populations (IPD-Work) consortium of European cohort studies. A collaboration of five studies was established at a workshop in London, UK on 8 November 2008, since when a further eight cohort studies have been included.

In this study, we pooled data from 13 prospective cohort studies (see Table 1 for full names): from Belgium (Belstress), Denmark (DWECS, IPAW, PUMA), Finland (FPS, HeSSup), France (Gazel), Germany (HNR), the Netherlands (POLS), Sweden (SLOSH, WOLF-N, WOLF-S) and the UK (Whitehall II). Details of the design, recruitment, measurements and ethical approval of the participating studies are presented in the Appendix S1. Participants with complete data on BMI, job strain, sex and age were included in these analyses, yielding a sample of 78 487 employed men and 83 259 employed women (mean age 43.7 years at study entry). Characteristics of these studies and the participants are shown in Table 1.

**Table 1:** Characteristics of participants in 13 European cohort studies

Study^a^ and country	Study years	Number of participants^b^	Number (%) of women	Mean age (range)	Mean (SD) BMI, kg m^−2^	Number (%) of cases of job strain
Belstress, Belgium	1994–1998	20 983	4928 (23)	45.5 (33–61)	26.1 (3.8)	3948 (19)
DWECS, Denmark	2000	5523	2567 (46)	41.8 (18–69)	24.6 (3.7)	1224 (22)
FPS, Finland	2000–2002	46 933	37 844 (81)	44.6 (17–65)	25.0 (4.1)	7641 (16)
Gazel, France	1997	11 259	3101 (28)	50.3 (43–58)	25.4 (3.5)	1630 (14)
HeSSup, Finland	1998	16 355	9067 (55)	39.6 (20–54)	24.9 (3.9)	2857 (17)
HNR, Germany	2000–2003	1823	742 (41)	53.4 (45–73)	27.4 (4.4)	221 (12)
IPAW, Denmark	1996–1997	1965	1305 (66)	41.3 (18–68)	24.2 (3.8)	339 (17)
POLS, the Netherlands	1997–2002	23 836	9891 (41)	38.3 (15–85)	24.4 (3.7)	3829 (16)
PUMA, Denmark	1999–2000	1774	1456 (82)	42.6 (18–69)	24.5 (3.9)	266 (15)
SLOSH, Sweden	2006 and 2008	10 698	5762 (54)	47.6 (19–68)	25.4 (3.9)	2103 (20)
Whitehall II, UK	1985–1988	10 262	3397 (33)	44.4 (34–56)	24.6 (3.5)	1440 (14)
WOLF-N, Sweden	1996–1998	4692	772 (16)	44.1 (19–65)	26.2 (3.6)	599 (13)
WOLF-S, Sweden	1992–1995	5643	2427 (43)	41.5 (19–70)	24.6 (3.6)	913 (16)
Total	1985–2008	161 746	83 259 (51)	43.7 (15–85)	25.1 (3.8)	27 010 (17)

BMI, body mass index.

a Study acronyms: DWECS, Danish Work Environment Cohort Study; FPS, Finnish Public Sector Study; HeSSup, Health and Social Support; HNR, Heinz Nixdorf Recall Study; IPAW, Intervention Project on Absence and Well-being; POLS, Permanent Onderzoek Leefsituatie; PUMA, Burnout, Motivation and Job Satisfaction study; SLOSH, Swedish Longitudinal Occupational Survey of Health; WOLF, Work, Lipids, Fibrinogen (N = Norrland, S = Stockholm).

b Individuals with complete data on job strain, age, sex and BMI.

### Assessment of BMI

Body mass index was calculated using the usual formula: weight in kilograms divided by height in metres squared. Participants with missing values for weight or height were excluded (*n* = 2220; 1.4% of all participants). To avoid a few potentially unreliable measurements unduly affecting the results, participants with BMI values <15 or >50 kg m^−2^ were excluded from the analysis (*n* = 100; 0.1%). We classified BMI into five categories according to World Health Organization (WHO) recommendations [22]. Participants with a BMI < 18.5 kg m^−2^ were categorized as underweight, those with a BMI between 18.5 and <25 kg m^−2^ were classified as normal weight, those with a BMI between 25 and <30 kg m^−2^ as overweight and, following the WHO international classification of adult obesity [22], we included two subcategories of obesity: class I (BMI 30 to <35 kg m^−2^) and class II and III combined (BMI ≥ 35 kg m^−2^).

### Definition of job strain

According to the job strain model – the most widely tested model of work stress – job strain arises when an employee simultaneously has high psychological job demands and a low level of control over work [23]. In the included studies, job strain was assessed using participant-completed questionnaires. All questions within the job demand and job control scales required responses in Likert-type formats. Mean response scores for job demand items and for job control items were calculated for each participant. An unfavourable (high) level of *job demand* was denoted by a score *above* the study-specific median, whereas an unfavourable (low) level of *job control* was defined as a score *below* the study-specific median score. We defined *job strain* as this combination of the two variables (i.e. a high level of job demand and a low level of job control). All other combinations of job demand and control, including levels equal to the median values, were defined as nonjob strain. Participants with missing data on more than half of the items of job demand or job control were excluded (*n* = 1714; 1.1%).

### Covariates

Covariates included in the analyses were age, sex, socio-economic status (SES; high, intermediate and low) and smoking status (current smoker versus nonsmoker). Participants with missing values for either age or sex were excluded from all analyses (*n* = 367; 0.2%). A more detailed description of the assessment of covariates is presented in the Appendix S1.

### Statistical analyses

We examined individual-level data from nine studies. For a further four, we provided syntax and instructions for statistical analysis, as the study investigators chose to carry out their own analyses. One-stage and two-stage meta-analyses of individual participant data [24–26] were performed. In the cross-sectional analysis, we used two-stage meta-analysis to include all cohort studies irrespective of whether individual-level or aggregate data were available from the study.

For each study, effect estimates and their standard errors were estimated using logistic regression (the first stage); these study-specific results were then pooled using random-effects meta-analysis (the second stage) [27]. We calculated summary odds ratios and their 95% confidence intervals (CIs) for job strain in individuals who were categorized as underweight, overweight or obese (classes I and II/III), compared with individuals of normal weight. We adjusted the odds ratios for sex, age, SES and smoking. To test the associations between BMI and both job demand and job control, we computed summary mean difference in demand and control scores between BMI categories using linear regression. Heterogeneity amongst study-specific estimates was assessed using the *I*^2^ statistic [28]. In a sensitivity analysis, we ran the analyses separately for studies in which individual-level data were available for pooled analysis. Additionally, to examine measurement method as a source of heterogeneity, we ran these analyses separately for studies with measured height and weight and for those with self-reported values.

In order to examine subgroup differences and longitudinal associations, we used a one-stage meta-analysis pooling all available individual-level data into one data set. We tested for possible interactions of BMI category, sex and age group (>50 vs. ≤50 years) by including an interaction term (BMI*covariate) in the model using a mixed-effects logistic regression model with study as the random effect. In four studies (Belstress, FPS, HeSSup and Whitehall II), BMI and job strain components had been re-measured approximately 4 years apart thus allowing us to examine the longitudinal associations between job strain and BMI categories in this subgroup of cohorts. To define job strain at follow-up, we used the same study-specific cut-off points that were used at baseline. These studies allowed us to examine a series of subsidiary questions. (i) Does exposure to job strain predict obesity amongst nonobese participants, and is an association with obesity stronger for those with repeated exposure to job strain (test of a dose–response association)? (ii) Are both weight gain (change from nonobese to obese between baseline and follow-up) and weight loss (change from obese to nonobese during the same period) related to the onset of job strain at follow-up? (iii) Does obesity at baseline predict the onset of job strain at follow-up (test of reverse causation)? (iv) Does SES at baseline predict obesity and job strain at follow-up and are the associations between job strain and obesity attenuated in a stratified analysis within the three strata of SES (test of the common cause hypothesis)?

Models were fitted with PROC GENMOD, PROC GLIMMIX and PROC MIXED in sas 9 or spss 17. The meta-analysis was conducted using r (version 2.11; library Meta, http://www.r-project.org). More details about statistical analysis can be found in the Appendix S1.

## Results

Amongst all participants, 86 429 (53.4%) were of normal weight (BMI 18.5–24.9 kg m^−2^), 2149 (1.3%) underweight (BMI < 18.5 kg m^−2^), 56 572 (35.0%) overweight (BMI 25.0–29.9 kg m^−2^), 13 523 (8.4%) obese class I (BMI 30–34.9 kg m^−2^) and 3073 (1.9%) obese classes II and III combined (BMI ≥ 35 kg m^−2^). A total of 27 010 (16.7%) participants reported job strain. Study-specific results are shown in Table 1.

### Job demand, job control and obesity

Figure S1 shows a forest plot of the mean differences in job demand score in each BMI category relative to the normal weight group. In an age- and sex-adjusted model (model 1), no association was observed between BMI category and job demand score. After further control for SES (model 2), there was some suggestion of a dose-related link, with higher job demand being associated with a higher risk of obesity, although all point estimates included zero. This positive relation was also seen in the longitudinal analysis of incident obesity (age-, sex- and SES-adjusted odds ratio for top versus bottom quintile 1.14, 95% CI 0.99–1.32, Table S1).

Figure S2 shows a corresponding forest plot for job control and BMI categories. In age- and sex-adjusted analyses (model 1), job control was slightly lower amongst underweight, overweight and obese participants compared with their normal-weight counterparts. However, after adding SES to the multivariable model, with the exception of the underweight group, all these differences were statistically nonsignificant (model 2).

### Job strain and obesity

**Fig. 1:**
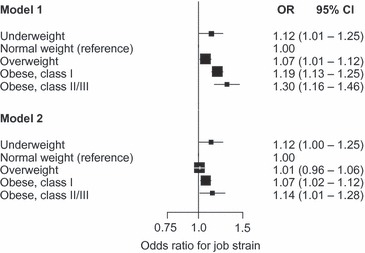
Summary estimates for the association between body mass index categories and high job strain. Model 1: adjusted for sex and age; model 2: additionally adjusted for socio-economic status (*n* = 161 746).

Figure 1 shows a forest plot of the random-effect summary odds ratios for job strain in each BMI category (study-specific results are provided in Fig. S3–S5). In an age- and sex-adjusted model (model 1), there was a suggestion of a ‘U’-shaped relation: the greatest risk of job strain was evident in the underweight and obese groups, whilst the risk was lowest in the normal-weight group. Thus, the odds ratio for job strain was 1.12 (95% CI 1.01–1.25) for underweight participants compared with those who were of normal weight. The corresponding odds ratios were 1.07 (95% CI 1.01–1.12) for overweight participants, 1.19 (95% CI 1.13–1.25) for class I obese participants and 1.30 (95% CI 1.16–1.46) for the combined class II and III obese groups. Adjustment for SES attenuated the odds ratios for the overweight and obese groups (model 2), but values remained statistically significant for the two obesity categories. Further adjustment for smoking had essentially no effect on these estimates.

### Longitudinal associations

Amongst the participants who were nonobese at baseline, low versus high SES at baseline was related to the risk of subsequent obesity, with an age- and sex-adjusted odds ratio of 1.54 (95% CI 1.35–1.76). Table 2 shows the longitudinal associations between job strain and obesity at follow-up in this population. These analyses are based on four cohort studies with a median (interquartile range) follow-up of 4 (1) years. Job strain at baseline alone or at both baseline and follow-up was not associated with obesity at follow-up. Similarly, in further analyses, change in BMI during follow-up did not differ between initially nonobese participants with and without job strain at baseline (age-, sex- and SES-adjusted mean difference in BMI change −0.02, 95% CI −0.06 to 0.02 kg m^−2^, *P* = 0.46), or between those with and without job strain at baseline and follow-up (mean difference −0.04, 95% CI −0.10 to 0.02 kg m^−2^, *P* = 0.22) (data not shown). However, new exposure to job strain at follow-up was associated with becoming obese at follow-up (odds ratio compared with no job strain at baseline and follow-up 1.18, 95% CI 1.02–1.36) (Table 2). When we examined this relationship within the three strata of SES, the results were essentially unchanged.

**Table 2:** Longitudinal association between job strain and incident obesity amongst nonobese participants in four studies with repeat data (*n* = 42 222)^a^

	Number of participants^b^	Number (%) of new cases of obesity	Obesity at follow-up OR (95% CI)^c^
Job strain at baseline			
No	35 715	1748 (4.9)	1.00 (reference)
Yes	6507	336 (5.2)	0.99 (0.88–1.12)
Job strain at baseline and at follow-up			
No and no	31 768	1518 (4.8)	1.00 (reference)
No and yes	3947	230 (5.8)	1.18 (1.02–1.36)^d^
Yes and no	3796	204 (5.4)	1.06 (0.92–1.24)
Yes and yes	2711	132 (4.9)	0.95 (0.79–1.14)

a Belstress, FPS, HeSSup and Whitehall II. Median follow-up 4 years.

b Participants who were of normal weight or overweight at baseline.

c Odds ratios are adjusted for age, sex, socio-economic status (SES).

d The corresponding age- and sex-adjusted odds ratios were 1.16 (95% CI 0.89–1.53) in the low-SES group (*n* = 7923), 1.18 (95% CI 0.97–1.43) in the intermediate-SES group (*n* = 23 151) and 1.25 (95% CI 0.86–1.83) in the high-SES group (*n* = 11 148).

Table 3 shows the longitudinal analysis relating BMI with job strain at follow-up amongst participants without job strain at baseline. Low SES at baseline was a strong predictor of job strain at follow-up (odds ratio 2.93, 95% CI 2.64–3.24), but baseline BMI categories were not associated with subsequent job strain (no support for the reverse causation hypothesis). Becoming obese was associated with a raised risk of job strain at follow-up (odds ratio 1.18, 95% CI 1.02–1.36). This was also evident within all strata of SES although all CI values included unity. Change from obese to nonobese was also associated with an increased odds of job strain at follow-up (odds ratio 1.31, 95% CI 1.03–1.68 compared with nonobese at baseline and follow-up), a finding replicated at low and intermediate levels of SES, although these analyses were restricted by low numbers (only five cases of incident job strain in the high-SES group).

**Table 3:** Longitudinal associations between body mass index (BMI) categories and job strain at follow-up amongst participants without job strain at baseline in four studies with repeat data (*n* = 39 970)^a^

	Number of participants^b^	Number (%) of new cases of job strain	Job strain at follow-up OR (95% CI)^c^
BMI category at baseline			
Underweight	446	54 (12.1)	1.05 (0.79–1.41)
Normal weight	22 701	2488 (11.0)	1.00 (reference)
Overweight	13 014	1459 (11.2)	1.04 (0.97–1.12)
Obese	3809	458 (12.0)	1.08 (0.96–1.20)
Obesity at baseline and at follow-up			
No and no	34 412	3771 (11.0)	1.00 (reference)
No and yes	1749	230 (13.2)	1.18 (1.02–1.36)
Yes and no	551	77 (14.0)	1.31 (1.03–1.68)^d^
Yes and yes	3258	381 (11.7)	1.03 (0.92–1.15)

a Belstress, FPS, HeSSup and Whitehall II. Median follow-up 4 years.

b Participants with no job strain at baseline.

c Odds ratios for BMI and obesity are adjusted for age, sex and socio-economic status (SES).

d The corresponding age- and sex-adjusted odds ratios were 1.34 (95% CI 0.86–2.10) in the low-SES group (*n* = 7192) and 1.47 (95% CI 1.07–2.02) in the intermediate-SES group (*n* = 21 402). There were only five new job strain cases amongst the high-SES participants who were obese at baseline but nonobese at follow-up.

### Sensitivity analyses

We found no statistical evidence to suggest that the cross-sectional association between job strain and obesity varied between particip ants younger and older than 50 years of age (*P* for interaction = 0.36) or between men and women (*P* for interaction = 0.35). Furthermore, the results described earlier remained largely unchanged after exclusion of the four studies that did not share individual-level data or when the analyses were performed separately for clinically measured versus self-reported BMI. Adjustment for the length of follow-up had essentially no effect on the longitudinal association estimates.

## Discussion

The aim of this analysis of pooled data from approximately 160 000 adults in 13 European studies was to describe the association between job strain and BMI in greater detail than has previously been possible. The results show a ‘U’-shaped association between the two factors, with the proportion of employees with job strain highest in the underweight and obese groups, and the lowest risk of job strain in individuals of normal weight. From two narrative reviews, based on studies with smaller numbers, the authors found no consistent cross-sectional association between work stress and BMI [29, 30]. However, in these analyses, the stressed and nonstressed participants were compared in terms of mean BMI, making it difficult to detect higher levels of stress amongst both underweight and obese individuals.

Our longitudinal analysis shows that changes in job strain and BMI category tend to co-occur. First, we found that change from no job strain at study baseline to job strain at follow-up is correlated with change from nonobese at baseline to obese at follow-up, a finding also apparent when we stratified analysis for each socio-economic group. Second, we found that change from no job strain at baseline to job strain at follow-up was also associated with reduction in weight (from obese to nonobese), again largely independently of SES. Thus, both weight gain and weight loss were associated with the onset of job strain, a finding which is consistent with the ‘U’-shaped cross-sectional association between job strain and BMI category.

We found little direct evidence to suggest that job strain is a causal risk factor for weight gain. First, the association was substantially reduced after adjustment for SES; second, baseline job strain did not predict change in BMI or the risk for obesity in longitudinal analysis. Finally, repeated measurements of job strain provided no evidence of dose–response associations between job strain and BMI or obesity. These findings are in agreement with those of previous studies. In a study-based meta-analysis of 8514 participants, Wardle and colleagues found no clear evidence for a longitudinal association between job strain and BMI (correlation coefficient 0.014, 95% CI −0.002 to 0.031, *P* = 0.09) [31]. This is in agreement with data from Japanese [32], Swiss [33], Swedish [34] and Finnish [35] studies which reported no association between job strain/work stress and change in adiposity. It has been suggested that the effect of job strain on change in BMI might differ between subgroups of individuals [10, 34], or the relationship may be limited to waist circumference [32]. However, BMI and waist circumference are strongly correlated [36] implying that a predictive association should also be seen for BMI if job strain was a strong predictor of waist circumference. Some studies have examined associations between other indicators of work stress (e.g. job insecurity or iso-strain) and weight change but with inconsistent findings [8, 15, 16, 33, 37].

Considering the reverse causation hypothesis, there was no evidence to suggest that obesity confers an increased risk of job strain. The fact that neither a direct causal effect nor the reverse causation hypothesis were supported by the results of our longitudinal analyses raises the possibility that common causes might underlie the apparent association between the onset of job strain and weight change. In cross-sectional age- and sex-adjusted analyses, the excess odds of job strain were approximately 20% in obese class I individuals and 30% for those in obese classes II/III; however, adjustment for SES attenuated these estimates to 7% and 14%, respectively. This attenuation suggests that socio-economic adversity is likely to at least partially explain the association between job strain and obesity. In the longitudinal analyses, similar associations between the onset of job strain and weight change were observed *within* socio-economic groups which means that these associations are unlikely to be solely explained by SES, but other, yet unknown, factors may also be involved. Further research is needed to confirm this. It may be that adverse life events and the onset of psychiatric disorders, particularly depressive symptoms, contribute to the association between the onset of job strain and weight gain, as these factors are known to affect weight control and reporting of job strain [38]. Previous research suggests a robust association between nonintentional weight loss, being underweight and increased mortality [39, 40], which is largely attributable to a pre-existing physical illness. This explanation might also apply in the present study with pre-existing physical morbidity potentially underlying the associations between weight loss, being underweight and job strain.

Our study has several important strengths. First, to our knowledge, this is the first study in which the association between BMI and job strain was studied across the entire BMI distribution; that is, including underweight individuals as well as two subcategories of obese individuals. Secondly, the analysis covers multiple study populations from several countries, increasing the generalizability of the findings. Given that the sample size was larger than in any prior study, the likelihood of random error influencing our results is also lower than in previous studies. Thirdly, we defined work stress based on the job strain model, which is the most widely used though not the only conceptualization in this area of research. However, some limitations should also be noted. Apart from SES and smoking, we did not examine the role of potential mediating or confounding factors. Despite data harmonization, variation in the assessment of job strain and SES between studies may have contributed to inaccuracy of the estimates. Data harmonization also meant that the measures of job strain and SES used in this study might not be optimally adjusted for the specific contexts of each participating study, potentially contributing to underestimation of the associations. On the other hand, using study-specific measurements, as in previous analyses, may introduce information bias and overestimation of the associations.

In summary, data from 13 European cohort studies show a cross-sectional ‘U’-shaped association between job strain and being either obese or underweight, and corresponding longitudinal associations between the onset of job strain and both weight gain and weight loss. As these associations were relatively modest in terms of absolute effect size and not necessarily causal, our data do not suggest that interventions to reduce job strain would be effective in combating obesity at a population level. However, early screening for job strain and obesity in the workplace may inform appropriate treatment strategies or lifestyle changes to prevent adverse health outcomes associated with these conditions, such as work disability and depressive disorders.

## Supporting Information

Additional Supplementary Informationmay be found in the online versionof this article:

**Figure S1.** Summary estimates for the association between BMI categories and job demand score.

**Figure S2.** Summary estimates for the association between BMI categories and job control score.

**Figure S3.** Study-specific meta-analysis of job strain amongst underweight(BMI < 18.5 kg m^−2^) versus normal weight (BMI 18.5–24.9 kg m^−2^) adults.

**Figure S4.** Study-specific meta-analysis of job strain amongst overweight (BMI 25.0–29.9 kg m^−2^) versus normal weight (BMI 18.5–24.9 kg m^−2^) adults.

**Figure S5.** Study-specific meta-analysis of job strain amongst obese (BMI ≥ 30 kg m^−2^) versus normal weight (BMI 18.5–24.9 kg m^−2^) adults.

**Table S1.** Longitudinal associations between job demand and job control quintiles and incident obesity in four studies with repeat data.

**Appendix S1.** Study design, recruitment of participants and measurements in the 13 participating studies.
