# Supplementary material for: Job strain in relation to body mass index: pooled analysis of 160 000 adults from 13 cohort studies
Source: J Intern Med. 2011 Dec 5;272(1):65–73. doi: 10.1111/j.1365-2796.2011.02482.x (PMC3437471; doi:10.1111/j.1365-2796.2011.02482.x)
Supplement: Supplementary file 1 [file joim0272-0065-SD1.docx]

**ONLINE APPENDIX: Supplementary Information**

**Study design, recruitment of participants and measurements in the 13 European studies included in the individual-participant meta-analyses of BMI and job strain.**

Details of the design, participant recruitment and measurements in the studies included in the meta-analyses are presented below. Participants were eligible for the meta-analyses if they were in employment and had available data on job strain.

Belstress

Belstress is a prospective cohort study to investigate the association between work-related stress and health outcomes. Between 1994 and 1998, a total of 21,419 people aged 35–59 years were recruited from the payroll records of 25 large companies or public administrations [1, 2]. Of these, 21,024 men and women had data on job strain and were eligible for our meta-analyses. The ethics committees of the University Hospital of Ghent and the Faculty of Medicine of the Université Libre de Bruxelles approved the Belstress study.

Danish Work Environment Cohort Study (DWECS)

DWECS is a split-panel survey of working aged Danish people. The cohort was established in 1990, when a simple random sample of men and women, aged 18–59 years, was drawn from the Danish population register. The participants have been followed up at 5-year intervals and data from the year 2000 were used for the IPD-Work consortium. That year, 11,437 individuals were invited to participate and 8583 agreed to do so [3, 4]. Of the 5606 individuals who were employed, 5574 had data on job strain and were eligible for our meta-analyses. DWECS was approved by and registered with the Danish Data protection agency (registration number: 2007-54-0059).

Finnish Public Sector (FPS)

The FPS study is a prospective cohort study comprising the entire public sector personnel of 10 towns (municipalities) and 21 hospitals in the same geographical areas. Participants, who were recruited from employers' records in 2000–2002, were individuals who had been employed in the study organisations for at least 6 months prior to data collection [5]. A total of 48,598 individuals responded to the questionnaire. Of these, 48,034 had data on job strain and were eligible for our meta-analyses. Ethical approval was obtained from the ethics committee of the Finnish Institute of Occupational Health.

Gazel

Gazel is a prospective cohort study of 20,625 employees (15,011 men and 5,614 women) of France's national gas and electricity company, Electricité de France-Gaz de France [6, 7]. Since the study baseline in 1989, participants (who were aged 35–50 years at baseline) have received by post an annual follow-up questionnaire to collect data on health and lifestyle, and individual, familial, social and occupational factors. Job strain was measured in Gazel in 1997; we treated this year as baseline for our analyses. The 11,448 individuals who participated at that time and had data on job strain were eligible for our meta-analyses. The Gazel study received approval from the national commission overseeing ethical data collection in France (Commission Nationale Informatique et Liberté).

Health and Social Support (HeSSup)

The HeSSup study is a prospective cohort study of a stratified random sample of the Finnish population in the following four age groups: 20–24, 30–34, 40–44 and 50–54 years. Subjects were identified from the Finnish population register and received by post an invitation to participate, along with a baseline questionnaire, in 1998. A follow-up questionnaire was sent to all participants still living in Finland in 2003 [8]. Job strain was measured in 1998 and of the 19,629 individuals who responded to the follow-up questionnaire, 17,102 had data on job strain and were eligible for our meta-analyses. The Turku University Central Hospital Ethics Committee approved the study.

Heinz Nixdorf Recall (HNR)

The HNR study is a prospective population-based cohort study of individuals randomly selected from the mandatory lists of residence in the metropolitan Ruhr area in Germany. Details of the study methods have been described previously [9, 10]. Briefly, 4814 participants aged 45–75 years were enroled at study baseline in 2000–2003. Job stress measures and comprehensive medical data were collected during the baseline examination. For the present analyses, baseline job strain measures were available for 1841 employed men and women. The HNR study was approved by the institutional local ethics committees and a quality management system was applied according to European industrial norms (DIN EN ISO 9001:2000).

Intervention Project on Absence and Well-being (IPAW)

IPAW is a 5-year psychosocial work environment intervention study including 22 intervention and 30 control work places in three organisations (a large pharmaceutical company, municipal technical services and municipal nursing homes) in Copenhagen, Denmark [11, 12]. The baseline questionnaire was posted to all employees at the selected work sites between 1996 and 1997. Of the 2721 employees who worked at the 52 IPAW sites, 2068 men and women completed the baseline questionnaire. Psychological, social support and other interventions took place at 22 workplaces during 1996–1998 at the organisational and interpersonal level. Job strain was measured in 1996–1997 and the 2031 participants who had data on job strain were eligible for our meta-analyses. IPAW was approved by and registered with the Danish Data Protection Agency (registration number: 2000-54-0066).

Permanent Onderzoek Leefsituatie (POLS)

POLS is a series of annual cross-sectional health and lifestyle surveys of Dutch men and women [13]. The participants are a representative sample of the Dutch population, drawn form the Municipal Population Register. Only those individuals living in private households were included. The data were mostly collected using computer-assisted personal interviewing. At study baseline in 1997–2002, a total of 59,441 men and women participated in the surveys. Of these, 24,761 were in paid employment, aged 15–85 years and had job strain data available, and were therefore eligible for our meta-analyses. POLS was approved by the medical ethics committee of the Netherlands Organisation for Applied Scientific Research.

Burnout, Motivation and Job Satisfaction study (PUMA)

PUMA is an intervention study of burnout among employees in the human service sector [14]. Selection criteria for the participating organisations were: between 200 and 500 employees, occupational groups within each organisation were willing to participate and organisations had to commit to the entire 5-year study period. At study baseline in 1999–2000, a total of 1914 subjects agreed to participate. Of these, 1847 individuals had data on job strain and were eligible for our meta-analyses. PUMA was approved by the Scientific Ethical Committees in the counties in which the study was conducted and approved by and registered with the Danish Data Protection Agency (registration number: 2000-54-0048).

Swedish Longitudinal Occupational Survey of Health (SLOSH)

SLOSH is a follow-up study of individuals who participated in the Swedish Work Environment Survey (SWES) in 2003 or 2005. SWES, conducted biennially by Statistics Sweden (SCB), is based on a sample of gainfully employed people aged 16–64 years drawn from the Labour Force Survey (LFS). These individuals were first included in LFS through stratification by county, sex, citizenship and inferred employment status.

Data from the 2006 and 2008 data collection waves of SLOSH were used in the IPD-Work analyses. In both years, data were collected using postal self-completion questionnaires. In 2006, 5,985 individuals responded to the questionnaire. Of these, 5141 people worked at least 12 h per week and 5104 had data on job strain and were thus eligible for our meta-analyses [15]. In 2008, a further 6751 individuals responded to the questionnaire [16]. Of these, 5895 men and women worked at least 12 h per week and 5866 had data on job strain and were thus eligible for our meta-analyses. SLOSH was approved by the Regional Research Ethics Board in Stockholm.

Whitehall II

The Whitehall II study is a prospective cohort study set up to investigate socio-economic determinants of health. At study baseline in 1985–1988, a total of 10,308 civil service employees (6895 men and 3413 women) aged 35–55 years and working in 20 civil service departments in London were invited to participate in the study [17]. Job strain was measured at study baseline and 10,285 men and women had data on job strain and were eligible for our meta-analyses. The Whitehall II study protocol was approved by the University College London Medical School committee on the ethics of human research. Written informed consent was obtained at each wave of data collection.

Work, Lipids, and Fibrinogen (WOLF) Stockholm (S) and Norrland (N) studies

The WOLF-S study is a prospective cohort study of 5698 people (3239 men and 2459 women) aged 19–70 years and working in companies in Stockholm county [18]. WOLF-N is a prospective cohort study of 4718 participants aged 19–65 years working in companies in Jämtland and Västernorrland counties [19]. At study baseline, participants underwent a clinical examination and completed a set of health questionnaires. For WOLF-S, the baseline assessment was undertaken at 20 occupational health service units between 1992 and 1995 and for WOLF-N at 13 occupational health service units in 1996–1998. The Regional Research Ethics Board in Stockholm, and the ethics committee at the Karolinska Institutet, Stockholm, Sweden approved the study.

ASSESSMENT OF BMI

BMI (weight in kilograms divided by height in metres squared) was calculated from clinically measured weight and height in five studies (Belstress, HNR, Whitehall II, WOLF-N and WOLF-S) and self-reported measurements in eight studies (DWECS, FPS, Gazel, HeSSup, IPAW, POLS, PUMA and SLOSH). POLS was the only study in which weight and height were coded in categories; we used the category mean for calculating BMI in that cohort.

Participants with missing values for weight or height were excluded (*n*=2220; 1.4%). To avoid a few potentially unreliable measurements unduly affecting the results, participants with BMI values <15 or >50 kg/m^2^ were excluded from the analysis (*n*=100; 0.1%).

ASSESSMENT OF COVARIATES

Sex and age were either obtained from registers or recorded in a medical examination (DWECS, FPS, Gazel, HNR, IPAW, PUMA, SLOSH, WOLF-N and WOLF-S) or from a questionnaire (Belstress, HeSSup, POLS and Whitehall II). In addition, we assessed SES and smoking as these factors may be related to both BMI and stress. SES was obtained from recorded occupation in DWECS, FPS, Gazel, IPAW and PUMA, from self-reported occupation in Belstress, HNR, POLS, SLOSH, WOLF-N, WOLF-S and Whitehall II and from education level in HeSSup, and was classified as low (e.g., cleaners or maintenance workers), intermediate (e.g., registered nurses or technicians), or high (e.g., teachers or physicians). Self-employed participants or participants with missing data on SES (*n*=4342; 2.7%) were categorised in a separate group ‘others’ (rather than excluded from the analysis) in order to maintain identical numbers for analyses with and without SES data. Smoking status was self-reported in all studies and categorised as ‘current smoker’ versus ‘non-smoker’. Participants with missing values for sex or age were excluded from the analysis (*n*=367; 0.2%).

STATISTICAL ANALYSIS

We examined individual-level data from the following nine studies: Belstress, FPS, Gazel, HeSSup, HNR, SLOSH, Whitehall II, WOLF-N and WOLF-S. For a further four studies (DWECS, IPAW, POLS and PUMA), we provided syntax and instructions for statistical analysis, as the study investigators chose to carry out their own analyses. In the analyses of job demand and job control scales, the scales were first constructed by calculating the mean response scores from the individual questions. The values were then cohort-wise standardised to have a mean of zero and standard deviation of one. In this procedure the study-specific mean score was subtracted from each participant's individual score and then divided by study-specific score's standard deviation. Linear regression analysis was then performed using these standardised values. Study-specific linear and logistic regression analysis models were fitted with PROC GENMOD in SAS 9 (Belstress, DWECS, FPS, Gazel, HeSSuP, HNR, IPAW, PUMA, SLOSH, Whitehall II, WOLF-N and WOLF-S) or SPSS 17 (POLS). Mixed models based on pooled data were fitted with PROC GLIMMIX and PROC MIXED in SAS 9. The meta-analysis was conducted using R (version 2.11, library Meta, www.r-project.org).

REFERENCES FOR SUPPLEMENTARY INFORMATION

1 Pelfrene E, Vlerick P, Mak RP, De Smets P, Kornitzer M, De Backe G. Scale Reliability and Validity of the Karasek `Job Demand-Control-Support’ Model in the Belstress Study. *Work & Stress,* 2001; **15:** 297-313.

2 De Bacquer D, Pelfrene E, Clays E*, et al.* Perceived Job Stress and Incidence of Coronary Events: 3-Year Follow-up of the Belgian Job Stress Project Cohort. *Am J Epidemiol* 2005; **161:** 434-41.

3 Burr H, Bjorner JB, Kristensen TS, Tüchsen F, Bach E. Trends in the Danish Work Environment in 1990–2000 and Their Associations with Labor-Force Changes. *Scand J Work Environ Health* 2003; **29:** 270-9.

4 Feveile H, Olsen O, Burr H, Bach E. Danish Work Environment Cohort Study 2005: From Idea to Sampling Design. *Statistics in Transition* 2007; **8:** 441-58.

5 Kivimaki M, Lawlor DA, Smith GD, Kouvonen A, Virtanen M, Elovainio M, Vahtera J. Socioeconomic Position, Co-Occurrence of Behavior-Related Risk Factors, and Coronary Heart Disease: The Finnish Public Sector Study. *Am J Public Health* 2007; **97:** 874-9.

6 Goldberg M, Leclerc A, Bonenfant S, Chastang JF, Schmaus A, Kaniewski N, Zins M. Cohort Profile: The Gazel Cohort Study. *Int J Epidemiol* 2007; **36:** 32-9.

7 Zins M, Leclerc A, Goldberg M. The French Gazel Cohort Study: 20 Years of Epidemiologi Research. *Advances in Life Course Research* 2009; **14:** 135-46.

8 Korkeila K, Suominen S, Ahvenainen J, Ojanlatva A, Rautava P, Helenius H, Koskenvuo M. Non-Response and Related Factors in a Nation-Wide Health Survey. *Eur J Epidemiol* 2001; **17:** 991-9.

9 Schmermund A, Mohlenkamp S, Stang A*, et al.* Assessment of Clinically Silent Atherosclerotic Disease and Established and Novel Risk Factors for Predicting Myocardial Infarction and Cardiac Death in Healthy Middle-Aged Subjects: Rationale and Design of the Heinz Nixdorf Recall Study. Risk Factors, Evaluation of Coronary Calcium and Lifestyle. *Am Heart J* 2002; **144:** 212-8.

10 Stang A, Moebus S, Dragano N*, et al.* Baseline Recruitment and Analyses of Nonresponse of the Heinz Nixdorf Recall Study: Identifiability of Phone Numbers as the Major Determinant of Response. *Eur J Epidemiol* 2005; **20:** 489-96.

11 Nielsen M, Kristensen T, Smith-Hansen L. The Intervention Project on Absence and Well-Being (Ipaw): Design and Results from the Baseline of a 5-Year Study. *Work and Stress* 2002; **16:** 191-206.

12 Nielsen ML, Rugulies R, Christensen KB, Smith-Hansen L, Bjorner JB, Kristensen T. Impact of the Psychosocial Work Environment on Registered Absence from Work: A Two-Year Longitudinal Study Using the Ipaw Cohort. *Work & Stress* 2004; **18:** 323-35.

13 de Groot W, Dekker R. The Dutch System of Official Social Surveys. *EuReporting Working Paper*. Mannheim: Mannheim Centre for European Social Research. 2001.

14 Borritz M, Rugulies R, Bjorner JB, Villadsen E, Mikkelsen OA, Kristensen TS. Burnout among Employees in Human Service Work: Design and Baseline Findings of the Puma Study. *Scand J Public Health* 2006; **34:** 49-58.

15 Magnusson Hanson LL, Theorell T, Oxenstierna G, Hyde M, Westerlund H. Demand, Control and Social Climate as Predictors of Emotional Exhaustion Symptoms in Working Swedish Men and Women. *Scand J Public Health* 2008; **36:** 737-43.

16 Hasson D, Theorell T, Westerlund H, Canlon B. Prevalence and Characteristics of Hearing Problems in a Working and Non-Working Swedish Population. *J Epidemiol Community Health* 2010; **64:** 453-60.

17 Marmot MG, Smith GD, Stansfeld S*, et al.* Health Inequalities among British Civil Servants: The Whitehall Ii Study. *Lancet* 1991; **337:** 1387-93.

18 Peter R, Alfredsson L, Hammar N, Siegrist J, Theorell T, P. W. High Effort, Low Reward, and Cardiovascular Risk Factors in Employed Swedish Men and Women: Baseline Results from the Wolf Study. *J Epidemiol Community Health* 1998; **52:** 540-7

19 Alfredsson L, Hammar N, Fransson E*, et al.* Job Strain and Major Risk Factors for Coronary Heart Disease among Employed Males and Females in a Swedish Study on Work, Lipids and Fibrinogen. *Scand J Work Environ Health* 2002; **28:** 238-48.

**Table S1. Longitudinal associations between job demand and job control quintiles and incident obesity in four studies with repeat data^a^**

|  |  | **Number of participants^b^ (Number of cases^c^)** | **Model 1**  OR (95% CI) | **Model 2**  OR (95% CI) |
| --- | --- | --- | --- | --- |
| Job demand | | | | |
|  | Q1 | 9528 (458) | 1.00 (reference) | 1.00 (reference) |
|  | Q2 | 10,353 (526) | 1.05 (0.92 to 1.20) | 1.07 (0.94 to 1.22) |
|  | Q3 | 7287 (349) | 0.99 (0.86 to 1.15) | 1.03 (0.90 to 1.19) |
|  | Q4 | 8133 (398) | 1.02 (0.89 to 1.17) | 1.08 (0.94 to 1.24) |
|  | Q5 | 7145 (367) | 1.07 (0.93 to 1.24) | 1.14 (0.99 to 1.32) |
| Job control | | | | |
|  | Q5 | 10,744 (502) | 1.00 (reference) | 1.00 (reference) |
|  | Q4 | 8751 (401) | 0.98 (0.85 to 1.12) | 0.93 (0.81 to 1.06) |
|  | Q3 | 8819 (433) | 1.04 (0.92 to 1.19) | 0.95 (0.83 to 1.09) |
|  | Q2 | 6416 (336) | 1.13 (0.98 to 1.31) | 0.99 (0.86 to 1.16) |
|  | Q1 | 7716 (426) | 1.18 (1.03 to 1.35) | 0.99 (0.85 to 1.14) |
|  |  |  |  |  |

Model 1 is adjusted for age and sex and model 2 additionally for SES.

^a^ Belstress, FPS, HeSSup and Whitehall II. Median follow-up 4 years.

^b^ Normal and overweight participants at baseline.

^c^ Obese participants at follow-up.

**Figure S1. *Summary estimates for the association between BMI categories and job demand score (model 1: adjusted for sex and age; model 2: additionally adjusted for SES) (n=161,746)***

Mean difference (95% CI)

Model 1

Underweight

Normal weight (reference)

Overweight

Obese, class I

Obese, class II/III

-0.02

0.00

-0.01

-0.02

-0.01

(-0.08 to 0.04)

(-0.03 to 0.01)

(-0.06 to 0.02)

(-0.08 to 0.06)

Model 2

Underweight

Normal weight (reference)

Overweight

Obese, class I

Obese, class II/III

-0.3

-0.2

-0.1

0

0.1

0.2

0.3

-0.02

0.00

0.01

0.02

0.06

(-0.07 to 0.03)

(-0.01 to 0.03)

(-0.01 to 0.06)

(-0.02 to 0.13)

Lower Higher

Level of job demands

(mean difference)

**Figure S2. *Summary estimates for the association between BMI categories and job control score (model 1: adjusted for sex and age; model 2: additionally adjusted for SES) (n=161,746)***

Model 1

Underweight

Normal weight (reference)

Overweight

Obese, class I

Obese, class II/III

-0.11

0.00

-0.05

-0.13

-0.19

(-0.15 to -0.06)

(-0.07 to -0.03)

(-0.16 to -0.11)

(-0.23 to -0.14)

Mean difference (95% CI)

Model 2

Underweight

Normal weight (reference)

Overweight

Obese, class I

Obese, class II/III

-0.3

-0.2

-0.1

0

0.1

0.2

0.3

-0.10

0.00

0.00

-0.02

-0.05

(-0.13 to -0.06)

(-0.01 to 0.01)

(-0.04 to 0.01)

(-0.09 to 0.00)

Lower Higher

Level of job control

(mean difference)

**Figure S3. Study-specific meta-analysis of job strain among underweight (BMI <18.5 kg/m^2^) versus normal weight (BMI 18.5–24.9 kg/m^2^) adults.(Model adjusted for age- and sex.)**

95%CI

OR

**Random-effects model**

Belstress

DWECS

FPS

Gazel

HeSSup

IPAW

POLS

PUMA

SLOSH

Whitehall II

WOLF-S

0.75

1.0

1.5

Odds ratio for job strain

**1.12**

1.01

0.99

1.17

1.29

1.07

1.22

0.96

1.67

1.88

1.25

0.97

**(1.01 to 1.25)**

(0.68 to 1.50)

(0.60 to1.62)

(0.94 to 1.45)

(0.83 to 2.03)

(0.78 to 1.47)

(0.63 to 2.36)

(0.77 to 1.20)

(0.76 to 3.64)

(1.18 to 3.00)

(0.81 to 1.93)

(0.54 to 1.74)

Weight

**100%**

7.5%

4.9%

25.6%

6.0%

12.0%

2.8%

23.9%

2.0%

5.5%

6.4%

3.5%

**Figure S4. Study-specific meta-analysis of job strain among overweight (BMI 25.0–29.9 kg/m^2^) versus normal weight (BMI 18.5–24.9 kg/m^2^) adults. (Model adjusted for age and sex.)**

**Figure S5. Study-specific meta-analysis of job strain among obese (BMI >30 kg/m^2^) versus normal weight (BMI 18.5–24.9 kg/m^2^) adults. (Model adjusted for age and sex)**

**Random-effects model**

Belstress

DWECS n

FPS

Gazel

HeSSup

HNR

IPAW

POLS

PUMA

SLOSH

Whitehall II

WOLF-N

WOLF-S

0.75

1.0

1.5

OR

**1.07**

1.13

1.06

1.10

1.17

1.06

1.08

1.24

1.00

0.60

1.14

0.93

1.03

1.12

95% CI

**(1.01** **to 1.12)**

(1.04 to 1.22)

(0.92 to 1.23)

(1.04 to 1.16)

(1.03 to 1.32)

(0.96 to 1.16)

(0.76 to 1.51)

(0.94 to 1.63)

(0.92 to 1.08)

(0.43 to 0.84)

(1.02 to 1.27)

(0.82 to 1.05)

(0.85 to 1.24)

(0.95 to 1.32)

Weight

**100%**

11.9%

7.1%

14.0%

8.6%

10.7%

2.0%

2.8%

11.8%

2.0%

9.6%

8.3%

5.0%

6.2%

Odds ratio for job strain

Odds ratio for job strain

**Random-effects model**

Belstress

DWECS

FPS

Gazel

HeSSup

HNR

IPAW

POLS

PUMA

SLOSH

Whitehall II

WOLF N

WOLF S

0.75

1.0

1.5

OR

**1.22**

1.24

1.87

1.22

1.33

1.23

1.25

1.12

1.06

0.97

1.38

1.30

1.35

1.77

95% CI

**(1.16** **to 1.28)**

(1.12 to 1.37)

(1.07 to 3.29)

(1.13 to 1.31)

(1.11 to 1.60)

(0.94 to 1.60)

(0.89 to 1.77)

(0.73 to 1.73)

(0.92 to 1.21)

(0.62 to 1.53)

(1.05 to 1.82)

(0.80 to 2.12)

(0.96 to 1.92)

(0.83 to 3.78)

Weight

**100%**

22.8%

0.8%

42.7%

7.1%

3.4%

2.0%

1.3%

12.3%

1.2%

3.1%

1.0%

2.0%

0.4%
